# Subacute Thyroiditis in Active COVID-19 Infection: A Report of Two Cases With a Systematic Review of the Literature

**DOI:** 10.7759/cureus.52611

**Published:** 2024-01-20

**Authors:** Mohammad Ashraf Ganie, Haroon Rashid, Ajaz Qadir, Parvaiz A Koul

**Affiliations:** 1 Endocrinology and Metabolism, Sher-i-Kashmir Institute of Medical Sciences, Srinagar, IND; 2 Clinical Research, Sher-i-Kashmir Institute of Medical Sciences, Srinagar, IND; 3 Internal Medicine/Pulmonary Medicine, Sher-i-Kashmir Institute of Medical Sciences, Srinagar, IND

**Keywords:** thyroiditis, subacute thyroiditis, sars-cov-2, de quervain’s thyroiditis, covid-19

## Abstract

Subacute thyroiditis (SAT) is a self-limiting inflammatory condition of the thyroid gland with distinct symptoms and a predictable outcome. During the current COVID-19 pandemic, there have been multiple isolated reports of SAT either during the active viral illness or following recovery. Here, we report two such cases of COVID-19 infection presenting with SAT. A 65-year-old male presented with a two-week history of anterior neck pain, odynophagia, high-grade fever (38.9°C), sweating, palpitations, and tremulousness. At physical examination, the patient presented with a slightly increased heart rate and a tender and enlarged thyroid on palpation. Laboratory examination showed high C-reactive protein levels, with elevated erythrocyte sedimentation rate, and thyroid function tests were suggestive of thyrotoxicosis. Ultrasonography showed a heterogeneous thyroid gland with ill-defined hypoechoic areas, and thyroid scintigraphy showed reduced uptake, confirming the diagnosis of SAT. In another case, a 52-year-old male presented with fever, cough, and myalgias, and was diagnosed with mild COVID-19 pneumonia, and managed conservatively. After two weeks, the patient had a recurrence of high-grade fever, odynophagia, palpitations, and tremors. Examination revealed tachycardia, hyperhidrosis, and a tender and enlarged thyroid on palpation. Thyroid function tests revealed low thyroid-stimulating hormone, with normal total T4 and total T3. Ultrasonography examination showed a heterogeneous thyroid gland with bilateral ill-defined hypoechoic areas. In our systematic review, including 103 SAT cases, it has been suggested that SAT should be recognized as an uncommon extra-pulmonary clinical manifestation of COVID-19 infection and clinicians need to be aware of the association. Pending larger multicentric studies, management of the condition has to be on a case-by-case basis.

## Introduction

Subacute thyroiditis (SAT), also known as subacute granulomatous, subacute non-suppurative, giant cell, painful, or de Quervain’s thyroiditis, is a self-limiting disorder, typically characterized by a painful tender thyroid gland along with systemic symptoms such as palpitations, tremor, fever, malaise, and anorexia [1]. Predominantly the disorder of women, SAT is reported to occur in 12.1 cases per 100,000 persons per year [2]. SAT generally manifests in three phases, i.e., thyrotoxic, hypothyroid, and recovery phase [1], and the commonest lab abnormalities are elevated erythrocyte sedimentation rate (ESR), C-reactive protein (CRP), T3, and T4, and low thyroid-stimulating hormone (TSH) [1]. The disorder, although self-limiting, can rarely cause persistent hypothyroidism [3,4].

A host of viral infections, including coxsackievirus [5], mumps [6], Epstein-Barr virus [7], cytomegalovirus [8], and influenza virus [1,9-12], have been incriminated in the causation of SAT. Initially, it was thought that this disease is common in summers [13], but other studies have shown it to be equally distributed throughout the season [4,14]. Over the last two years, during the unprecedented pandemic of coronavirus disease 2019 (COVID-19), a number of case reports of SAT have appeared suggesting that severe acute respiratory syndrome coronavirus 2 (SARS-CoV-2) viral infection acts as a trigger of SAT, which may present either during the infection or after it has resolved [2].

COVID-19 is a highly contagious disease caused by the SARS-CoV-2 [15], a positive-sense, single-stranded, enveloped RNA virus belonging to the beta-coronavirus family. SARS-CoV-2 is phylogenetically related to SARS-CoV-1, the virus that causes severe acute respiratory syndrome (SARS). During the SARS-CoV-1 outbreak, the histopathology of the thyroid gland of infected individuals demonstrated the destruction of epithelial cells, parafollicular cells, and follicular epithelial cells [16]. The main mechanism proposed for this thyroid injury is extensive apoptosis of cells rather than cell necrosis or inflammatory infiltration. Although SARS-CoV-1 results in severe infection, its effect on thyroid is believed to be less severe than that of SARS-CoV-2 [16].

Early in the current SARS-CoV-2 pandemic, several reports of SAT and cases of thyroid dysfunction were reported [17-19] and this led to a notion that SAT was an underestimated manifestation of COVID-19 [20-24]. As per a recent systematic review, thyroid dysfunction in COVID-19 patients in intensive care units may partly be accounted for by non-thyroidal illness syndrome. Furthermore, several studies have shown that thyroid dysfunction does not increase the risk of SARS-CoV-2 infection and hence patients with thyroid dysfunction may not need any COVID-19-adapted follow-up [25].

We herewith report two cases of SAT temporally associated with acute COVID-19 infection. Besides, we have systematically reviewed the literature to describe clinical characteristics, treatment requirements, resolution rates, and outcomes of SAT in COVID-19 patients.

## Case presentation

Case 1

**Figure 1:**
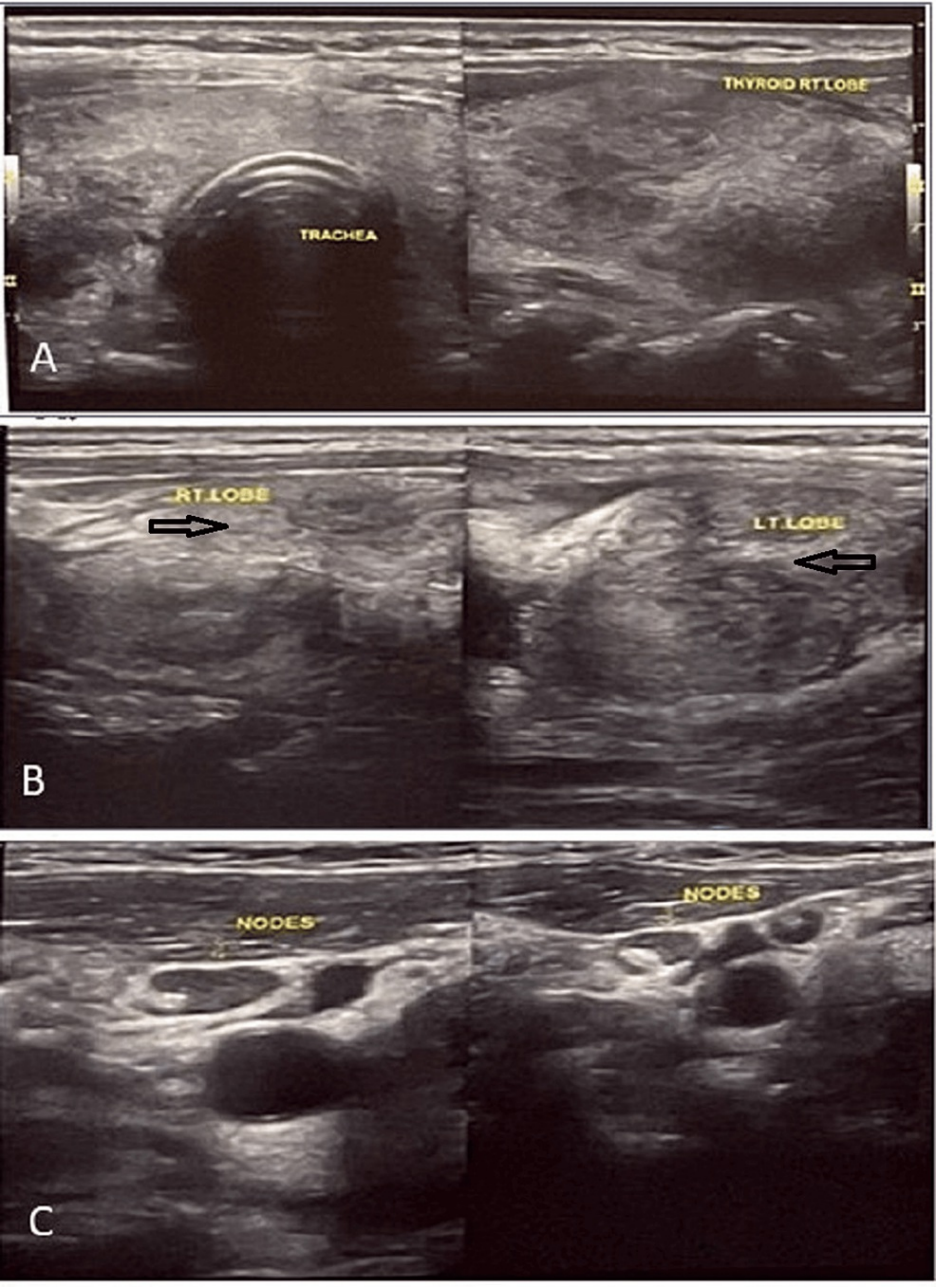
Ultrasonographic images of thyroid lobes. A and B: Enlarged isthmus, right and left lobe of the thyroid gland with coarse heterogeneous echo texture. C. Enlarged cervical lymph nodes with maintained fatty hilum.

AB, a 65-year-old male, presented with a two-week history of anterior neck pain, odynophagia, high-grade fever (38.9°C), sweating, palpitations, and tremulousness. In the preceding week, the patient had a history of cough, myalgias, fever, and fatigue. A rapid antigen test was positive for COVID-19 and family members exhibited a similar illness. The patient had received two doses of COVISHIELD (Oxford-AstraZeneca vaccine) and the last dose was given seven months back. On current examination, the patient was conscious and oriented with a heart rate of 100 per minute, blood pressure of 120/80mmHg, and BMI of 27.99 kg/m2. The thyroid gland was enlarged (WHO grade II) and tender, with no bruit. Laboratory tests on admission showed a high CRP level, with elevated ESR and thyroid function tests, suggestive of thyrotoxicosis (a low TSH, elevated total T4, and elevated total T3) (Table 1). Thyroid-peroxidase antibody (TPO-Ab) was negative and an ultrasound of the neck showed a heterogeneous thyroid gland (right lobe: 2.1 × 2.6 × 4.5 cm; left lobe: 2.2 × 2.6 × 4.2 cm) with ill-defined hypoechoic areas and small cervical lymph nodes (Figure 1). Radiography of the chest revealed bilateral ground glass opacities and the oxygen saturation was normal. Thyroid scintigraphy showed reduced uptake, confirming the diagnosis of subacute thyroiditis. The patient was treated with ibuprofen 400 mg twice daily and propranolol 40 mg once a day for one week. Since the symptoms continued, the patient was put on prednisolone 60 mg daily for one week, followed by tapering of the dose over eight weeks. Although repeat thyroid function after six weeks revealed normal total T4 (10.43 μg/dl), total T3 (121.2 ng/dl), and TSH (3.780 mIU/mL), the patient developed dysglycemia, which was managed with a combination of teneligliptin 20 mg and metformin 1 gm daily.

**Table 1:** Laboratory results of the cases. CRP: C-reactive protein; ESR: erythrocyte sedimentation rate; TSH: thyroid-stimulating hormone; T4: tetraiodothyronine; T3: triiodothyronine; TPO-Ab: thyroid peroxidase antibody.

Patient	Laboratory parameters	Patient values	Reference ranges
Case 1 (65 years/male)	CRP (mg/L)	148.24	<10
	ESR (mm/h)	86	<15
	TSH (mIU/L)	<0.03	0.2-4.5
	T4 (μg/dL)	19.07	4-11
	T3 (ng/dL)	230.02	75-195
	TPO-Ab (U/mL)	0.31	<10
Case 2 (52 years/male)	CRP (mg/L)	8	<10
	TSH (mIU/L)	<0.17	0.2-4.5
	T4 (μg/dL)	10.9	4-11
	T3 (ng/dL)	126	75-195

Case 2

XY, a 52-year-old male, presented with fever, cough, and myalgias with a similar illness in all the family members and was detected positive by real-time polymerase chain reaction (RT-PCR) on the nasopharyngeal swab for SARS-CoV-2. The patient had received two doses of COVISHIELD (Oxford-AstraZeneca vaccine) and the last dose was given on 22nd April 2021, three months before the index presentation. In view of chest X-ray findings (bilateral ground glass opacities), the patient was diagnosed with mild COVID-19 pneumonia and managed conservatively. Two weeks following this illness, the patient had a recurrence of high-grade fever, odynophagia, palpitations, and tremors. Examination revealed tachycardia (pulse rate = 102 beats/minute), hyperhidrosis grade II, and tender thyromegaly with no orbitopathy or dermopathy. Thyroid function tests revealed low TSH, with normal total T4 and total T3. CRP level was normal (Table 1). Ultrasonography examination showed a heterogeneous thyroid gland (right lobe: 45 × 31 mm; left lobe: 26 × 12mm) with bilateral ill-defined hypoechoic areas. The patient was treated with propranolol 40 mg once a day and prednisolone 50 mg daily for one week followed by tapering over four weeks. The symptoms resolved in six weeks and repeat thyroid function was normalized by then.

## Discussion

**Figure 2:**
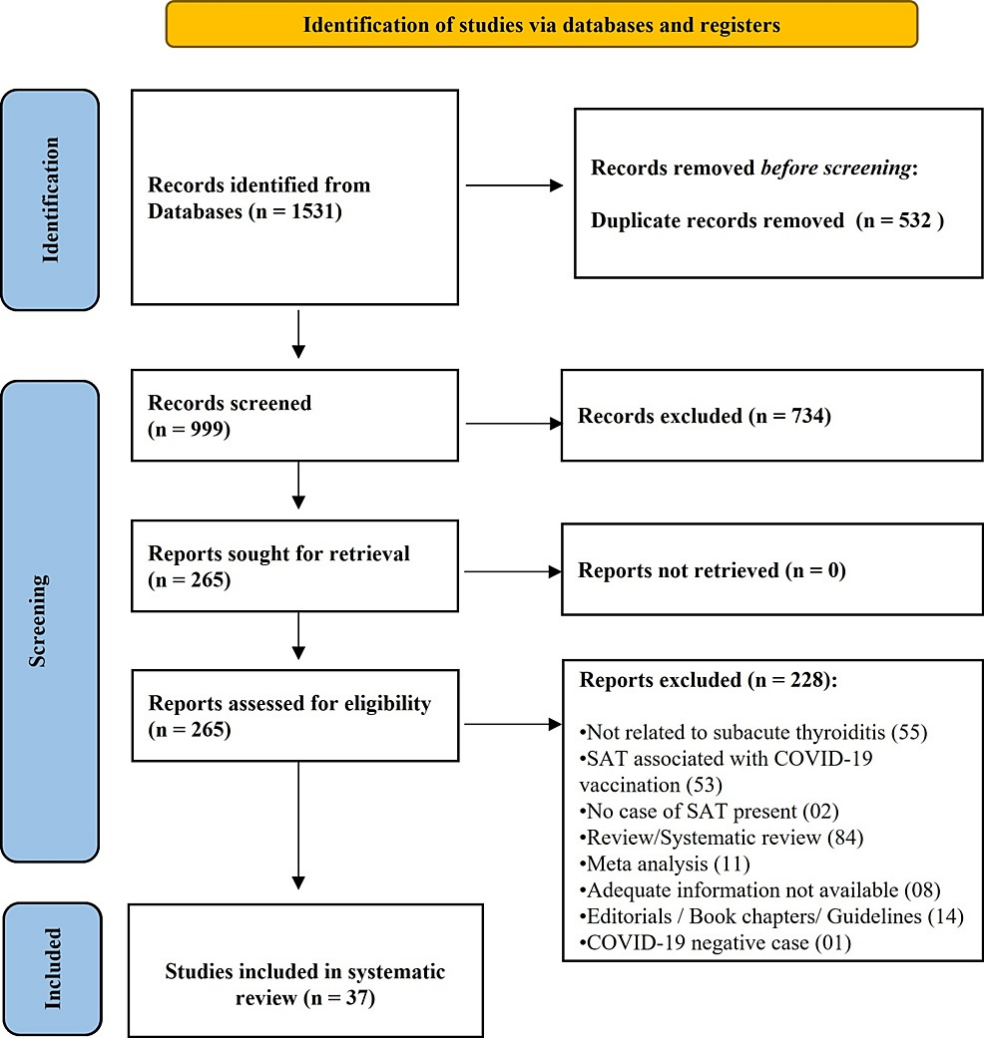
Flow chart to illustrate the process by which articles were selected or rejected for inclusion in the study. SAT: subacute thyroiditis.

A systematic review of the literature was performed using Preferred Reporting Items for Systematic Reviews and Meta-Analyses (PRISMA) checklist guidelines [26] and was prospectively registered on the International Prospective Register of Systematic Reviews (PROSPERO; CRD42022310160). A comprehensive literature search was carried out in seven different databases until May 5, 2022, for articles reporting SAT in COVID-19 patients using broad key terms: (COVID OR COVID-19 OR SARS-CoV-2 OR coronavirus OR COVID-19 vaccination) and (thyroiditis OR subacute thyroiditis OR thyrotoxicosis). In all, there were 1,531 citations: 689 from PubMed, 620 from ScienceDirect, 143 from the WHO COVID-19 database, 37 from Scopus, 25 from Embase, and 17 from The Cochrane Library, and among these 532 duplicate cases were removed. Titles and abstracts of 999 studies were screened and a total of 734 records were excluded. The remaining 265 articles met the criteria for full-text review. The criteria for inclusion of the published studies of any design (including case reports) were COVID-19-associated SAT, and the exclusion criteria were: (1) studies not related to SAT; (2) studies not providing sufficient data; (3) studies without results; (4) commentaries, guidelines, editorials, book chapters, reviews, and meta-analysis; (5) animal studies; (6) COVID-19-negative SAT cases; (7) studies without any SAT case/patient. After applying exclusion criteria, out of 265 full texts, 228 were excluded and 37 articles were included for final qualitative analysis. The selection process of the studies is displayed in the flow diagram (Figure 2). The finally selected 37 articles included 32 case reports, one case series (11 SAT cases), one cross-sectional study (18 SAT cases), one prospective observational study (12 SAT cases), one retrospective observational study (five SAT cases), and one retrospective-prospective cohort (11 SAT cases). The quality of all selected studies/reports was assessed by two independent reviewers using the Joanna Briggs Institute's (JBI) Critical Appraisal Checklist for Studies, including the risk of bias in studies/reports [27]. The percentage of "yes" scores ranged from 50% to 100% in all of the articles included in the study, indicating a low to moderate risk of bias.

A total of 103 cases of COVID-19 with SAT were identified, and the number of cases in the studies ranged from one to 18. Table 2 summarizes the key demographic and clinical characteristics of each enrolled patient. The age of the patients ranged from 18 to 73 years with a median of 41 years (IQR: 34-52), and out of the described 103 patients, 79 were females (76.7%). Of the included 37 studies that were published from 17 countries, eight (21.6%) were from the United States of America, five (13.5%) from Italy, four (10.8%) from Spain, three (8.1%) each from Turkey and India, and two (5.4%) each from Iran and Mexico. The cases were reported from Europe (n = 47, 45.6%), Asia (n = 43, 41.7%), North America (n = 10, 9.7%), and South America (n = 3, 2.9%).

**Table 2:** Demographics and clinical presentation of COVID-19-associated subacute thyroiditis. SAT: subacute thyroiditis; COVID-19: coronavirus disease 2019; RT-PCR: reverse transcription polymerase chain reaction; IgG: immunoglobulin G.

Author	Year	Country	Study design	N	Age	Gender	COVID-19 diagnosis	Clinical presentation of COVID-19	Onset of SAT (days)	Presence of goiter	Neck pain	Fever	Palpitations	Tremors
Bahçecioğlu et al. (P1) [28]	2022	Turkey	Prospective cohort	1	48	Female	RT-PCR	Mild COVID-19 symptoms	42	NO	YES	NO	NO	NO
Bahçecioğlu et al. (P2) [28]	2022	Turkey	Prospective cohort	1	40	Male	RT-PCR	Mild COVID-19 symptoms	91	NO	YES	NO	YES	NO
Bahçecioğlu et al. (P3) [28]	2022	Turkey	Prospective cohort	1	40	Male	RT-PCR	Mild COVID-19 symptoms	84	NO	YES	YES	NO	NO
Bahçecioğlu et al. (P4) [28]	2022	Turkey	Prospective cohort	1	56	Male	RT-PCR	Mild COVID-19 symptoms	56	NO	YES	NO	NO	NO
Bahçecioğlu et al. (P5) [28]	2022	Turkey	Prospective cohort	1	54	Female	RT-PCR	Mild COVID-19 symptoms	21	NO	YES	NO	NO	NO
Bahçecioğlu et al. (P6) [28]	2022	Turkey	Prospective cohort	1	52	Male	RT-PCR	Mild COVID-19 symptoms	49	NO	YES	NO	YES	NO
Bahçecioğlu et al. (P7) [28]	2022	Turkey	Prospective cohort	1	37	Male	RT-PCR	Mild COVID-19 symptoms	168	NO	YES	NO	NO	NO
Bahçecioğlu et al. (P8) [28]	2022	Turkey	Prospective cohort	1	51	Male	RT-PCR	Mild COVID-19 symptoms	168	NO	YES	NO	NO	NO
Bahçecioğlu et al. (P9) [28]	2022	Turkey	Prospective cohort	1	36	Female	RT-PCR	Asymptomatic for COVID-19	NA	NO	YES	NO	NO	NO
Bahçecioğlu et al. (P10) [28]	2022	Turkey	Prospective cohort	1	53	Female	RT-PCR	Mild COVID-19 symptoms	Concurrent	NO	YES	YES	NO	NO
Bahçecioğlu et al. (P11) [28]	2022	Turkey	Prospective cohort	1	50	Male	RT-PCR	Mild COVID-19 symptoms	Concurrent	NO	YES	NO	YES	NO
Bahçecioğlu et al. (P12) [28]	2022	Turkey	Prospective cohort	1	48	Female	RT-PCR	Moderate COVID-19 symptoms	Concurrent	NO	YES	YES	NO	NO
Álvarez Martín et al. [29]	2020	Spain	Case report	1	46	Female	Positive IgG	Asymptomatic for COVID-19	NA	YES	YES	YES	NO	YES
Abreu et al. (P1) [30]	2021	Brazil	Case report	1	34	Female	RT-PCR	Mild COVID-19 symptoms	28	NO	YES	YES	NO	NO
Abreu et al. (P2) [30]	2021	Brazil	Case report	1	34	Female	RT-PCR	Asymptomatic for COVID-19	10	NO	NO	NO	NO	NO
Abreu et al. (P3) [30]	2021	Brazil	Case report	1	39	Female	RT-PCR	Mild fever and anosmia	26	NO	YES	YES	NO	NO
Asfuroglu et al. [19]	2020	Turkey	Case report	1	41	Female	RT-PCR	Asymptomatic for COVID-19	Concurrent	NO	YES	YES	NO	NO
Ashraf et al. [31]	2021	USA	Case report	1	58	Female	RT-PCR	Mild COVID-19 symptoms	12	NO	NO	YES	NO	NO
Asimi & Dzuvo [32]	2021	Bosnia	Case series	11	30-45	Female	RT-PCR	Mild COVID-19 symptoms	NA	YES	YES	YES	YES	YES
Barahona et al. [33]	2021	Spain	Case report	1	52	Male	RT-PCR	Mild COVID-19 symptoms	NA	NO	NO	NO	NO	NO
Brancatella et al. [34]	2021	Italy	Cross-sectional study	18	34 ± 14	Female	NA	Mild COVID-19 symptoms (78% of patients)	29 (median)	NA	16 (89%)	17 (94%)	NO	NO
Brancatella et al. (P1) [17]	2020	Italy	Case report	1	38	Female	RT-PCR	Mild COVID-19 symptoms	16	NO	YES	YES	YES	NO
Brancatella et al. (P2) [17]	2020	Italy	Case report	1	29	Female	Positive IgG	Mild COVID-19 symptoms	30	NO	YES	YES	YES	NO
Brancatella et al. (P3) [17]	2020	Italy	Case report	1	29	Female	RT-PCR	Mild COVID-19 symptoms, anosmia	36	YES	YES	NO	YES	NO
Brancatella et al. (P4) [17]	2020	Italy	Case report	1	46	Female	RT-PCR	Mild COVID-19 symptoms, anosmia	20	NO	YES	YES	YES	NO
Brancatella et al. [35]	2020	Italy	Case report	1	18	Female	RT-PCR	Mild COVID-19 symptoms	19	NO	YES	YES	YES	NO
Campos-Barrera et al. [2]	2020	Mexico	Case report	1	37	Female	RT-PCR	Mild COVID-19 symptoms, odynophagia, and anosmia	30	NO	YES	NO	NO	NO
Chakraborty et al. [36]	2020	India	Case report	1	58	Male	RT-PCR	Mild COVID-19 symptoms	4	YES	YES	YES	YES	NO
Chong et al. [37]	2021	USA	Case report	1	37	Male	RT-PCR	Mild COVID-19 symptoms	30	NO	YES	NO	YES	YES
Das & Fatima [38]	2021	Pakistan	Case report	1	33	Male	RT-PCR	Mild COVID-19 symptoms	7	NO	YES	YES	YES	NO
Davoodi et al. [39]	2021	Iran	Case report	1	33	Male	RT-PCR	Mild COVID-19 symptoms	8	NO	YES	YES	YES	NO
de la Higuera López-Frías et al. [40]	2021	Spain	Case report	1	36	Female	Positive IgG	Mild COVID-19 symptoms	45	NO	YES	YES	NO	NO
De San Juan et al. [41]	2020	Philippines	Case report	1	47	Female	RT-PCR	Right lower lobe pneumonia	NA	YES	YES	NO	NO	NO
Dworakowska et al. [42]	2021	United Kingdom	Case report	1	57	Female	NA	Mild COVID-19 symptoms	84	YES	YES	NO	YES	YES
Feghali et al. [43]	2021	USA	Case report	1	41	Female	NA	Mild COVID-19 symptoms	42	NO	NO	NO	YES	NO
Ippolito et al. [44]	2020	Italy	Case report	1	69	Female	RT-PCR	Pneumonia	5	YES	NO	NO	YES	NO
Khatri et al. [45]	2021	USA	Case report	1	41	Female	RT-PCR	Mild COVID-19 symptoms	14	NO	YES	YES	YES	YES
Mathews et al. [46]	2021	USA	Case report	1	67	Male	RT-PCR	Pneumonia	Concurrent	NO	NO	NO	NO	NO
Mattar et al. [47]	2020	Singapore	Case report	1	34	Male	RT-PCR	Mild COVID-19 symptoms	9	YES	YES	NO	YES	NO
Mehmood et al. [48]	2020	USA	Case report	1	29	Female	RT-PCR	Mild COVID-19 symptoms	49	NO	YES	YES	YES	YES
Osorio Martínez et al. [49]	2021	Mexico	Case report	1	64	Male	RT-PCR	Moderate COVID-19 symptoms	21	NO	NO	NO	NO	NO
Ramsay et al. [50]	2021	USA	Case report	1	51	Female	NA	Mild COVID-19 symptoms	23	NO	YES	YES	YES	YES
Seyed Resuli & Bezgal (P1) [51]	2021	Turkey	Retrospective cohort	1	32	Female	RT-PCR	NA	NA	NO	YES	NO	YES	NO
Seyed Resuli & Bezgal (P2) [51]	2021	Turkey	Retrospective cohort	1	25	Female	RT-PCR	NA	NA	NO	YES	NO	YES	NO
Seyed Resuli & Bezgal (P3) [51]	2021	Turkey	Retrospective cohort	1	45	Female	RT-PCR	NA	NA	NO	YES	YES	NO	NO
Seyed Resuli & Bezgal (P4) [51]	2021	Turkey	Retrospective cohort	1	29	Female	RT-PCR	NA	NA	NO	YES	NO	YES	NO
Seyed Resuli & Bezgal (P5) [51]	2021	Turkey	Retrospective cohort	1	21	Female	RT-PCR	NA	NA	NO	YES	YES	NO	NO
Ruano et al. [52]	2020	Spain	Case report	1	28	Female	RT-PCR	Mild COVID-19 symptoms	14	YES	YES	YES	YES	NO
Ruggeri et al. [18]	2020	Italy	Case report	1	43	Female	RT-PCR	Mild COVID-19 symptoms	42	YES	YES	YES	YES	YES
Sohrabpour et al. (P1) [53]	2021	Iran	Case report	1	26	Female	Positive IgG	Mild COVID-19 symptoms	30	NO	YES	YES	YES	NO
Sohrabpour et al. (P2) [53]	2021	Iran	Case report	1	37	Female	Positive IgG	Mild COVID-19 symptoms	30	NO	YES	YES	YES	NO
Sohrabpour et al. (P3) [53]	2021	Iran	Case report	1	35	Male	Positive IgG	Asymptomatic for COVID-19	30	NO	YES	YES	YES	NO
Sohrabpour et al. (P4) [53]	2021	Iran	Case report	1	41	Female	Positive IgG	Mild COVID-19 symptoms	30	NO	YES	YES	YES	NO
Sohrabpour et al. (P5) [53]	2021	Iran	Case report	1	52	Male	Positive IgG	Mild COVID-19 symptoms	30	NO	YES	YES	YES	NO
Sohrabpour et al. (P6) [53]	2021	Iran	Case report	1	34	Female	Positive IgG	Asymptomatic for COVID-19	30	NO	YES	YES	YES	NO
Sato et al. [54]	2021	Japan	Case report	1	31	Female	RT-PCR	Mild COVID-19 symptoms	14	NO	YES	YES	NO	NO
Semikov et al. (P1) [55]	2021	Russia	Case report	1	45	Female	RT-PCR	Pneumonia	Concurrent	NO	YES	YES	YES	NO
Semikov et al. (P2)	2021	Russia	Case report	1	40	Female	RT-PCR	Pneumonia	30	NO	YES	YES	YES	NO
Sherpa et al. [56]	2021	USA	Case report	1	73	Female	RT-PCR	Pneumonia	12	NO	NO	NO	NO	NO
Stasiak et al. (P1) [57]	2021	Poland	Case report	1	50	Male	RT-PCR	Mild COVID-19 symptoms	Concurrent	NO	YES	YES	NO	NO
Stasiak et al. (P2) [57]	2021	Poland	Case report	1	39	Female	RT-PCR	Mild COVID-19 symptoms	35	NO	YES	YES	NO	YES
Stasiak et al. (P3) [57]	2021	Poland	Case report	1	55	Female	RT-PCR	Mild COVID-19 symptoms	35	NO	YES	YES	NO	NO
Stasiak et al. (P4) [57]	2021	Poland	Case report	1	57	Female	RT-PCR	Mild COVID-19 symptoms	112	NO	YES	YES	NO	NO
Thimmaiah et al. [58]	2021	India	Case report	1	73	Male	NA	Mild COVID-19 symptoms	Concurrent	NO	NO	NO	YES	NO
Tjønnfjord et al. [59]	2021	Norway	Case report	1	40-50	Male	RT-PCR	Mild COVID-19 symptoms	21	NO	NO	YES	NO	NO
Mondal et al. [60]	2022	India	Retrospective-prospective cohort	1	48 (median)	Male: 4; female: 7	RT-PCR	Severe: 5, moderate: 5, asymptomatic: 1	26 (median)	NA	6 (54.5%)	5 (45.5%)	6 (54.5%)	5 (45.5%)

In 72 patients (69.9%), COVID-19 was diagnosed using RT-PCR and no information regarding testing was available in 21.4% of patients. COVID-19 was asymptomatic in seven cases (6.8%), with mild to moderate respiratory symptoms in 80 cases (77.7%), and severe in 11 cases (10.7%). Nineteen percent (20 cases) reported the presence of goiter prior to the onset of the symptoms. The onset of the timing of SAT diagnosis in relation to COVID-19 was mentioned only in 83 patients. Among the subjects where the information was available, the time interval between the start of COVID-19 illness and the appearance of SAT symptoms ranged from four to 168 days (median: 28, IQR: 12-36) in 73 (70.8%) cases. SAT occurred concurrently with COVID-19 among eight (7.8%) cases. Overall, 92.2% of patients had some symptoms suggestive of SAT with the most common ones as neck pain, fever, palpitations, and tremors. Neck pain was experienced by 86 (83.5%) patients, fever by 71 (68.9%), palpitations by 50 (48.5%), and tremors by 25 (24.3%) patients. Other clinical features included anxiety, agitation, insomnia, weight loss, excessive sweating, asthenia, and malaise. Hyperadrenergic symptoms (52.4%) were commoner than goiter (19%) and neck tenderness (49.5%).

Table 3 summarizes the laboratory findings and the results of thyroid-specific imaging performed on the included patients. Deranged thyroid function tests (T3, T4, and TSH) were seen in most of the patients, with each case having low TSH, and either high T3 or high T4 or both. Median TSH level (available in 51 out of 103 cases) was 0.01 (IQR: 0.003-0.04) mIU/L, median T4 (available in 55 cases) was 28.1 (IQR: 24.1-38.6) pmol/L, and median T3 (available in 42 cases) was 17.5 (IQR: 8.7-20.2) pmol/L. Anti-thyroid antibody testing (TPO-Ab) was reported in 56 patients and was detectable in only seven patients. Out of the 103 patients, elevated CRP was reported in 80 cases with a median level of 45.5 (IQR: 14.7 83) mg/L. Of the 80 cases where ESR was reported, 78 cases (72.8%) had an elevated value with a median of 65 (IQR: 46.5-83.5) mm/hr. Information regarding thyroid USG was available for 95 cases (92.2%) and all these cases had thyroid USG features suggestive of SAT. Sonographic features suggestive of SAT in the patients included patchy hypoechogenic areas (70%), enlarged thyroid gland (53.6%), and decreased vascularity (22%). Thyroid scintigraphy was performed in 39 (37.9%) patients, with the uptake being absent in nine cases, and reduced in 29 cases.

**Table 3:** Biochemical evaluation of thyroid function parameters, thyroid auto-antibodies, inflammatory markers, and results of thyroid-specific imaging in the included studies. CRP: C-reactive protein; ESR: erythrocyte sedimentation rate; TSH: thyroid-stimulating hormone; T4: tetraiodothyronine; T3: triiodothyronine; TPO-Ab: thyroid peroxidase antibody; NA: not available.

Author	Year	T3	T4	TSH	TPO-Ab	CRP	ESR	Ultrasonography	Thyroid scan
Bahçecioğlu et al. (P1) [28]	2022	High	High	Low	NA	High (54 mg/L)	High (49 mm/h)	Bilateral	NA
Bahçecioğlu et al. (P2) [28]	2022	High	High	Low	NA	High (49.6 mg/L)	Normal (17 mm/h)	Bilateral	NA
Bahçecioğlu et al. (P3) [28]	2022	High	High	Low	NA	High (80 mg/L)	High (54 mm/h)	Unilateral	NA
Bahçecioğlu et al. (P4) [28]	2022	High	High	Low	NA	High (40 mg/L)	Normal (11 mm/h)	Bilateral	NA
Bahçecioğlu et al. (P5) [28]	2022	High	High	Low	NA	High (51 mg/L)	High (59 mm/h)	Unilateral (the left lobe)	NA
Bahçecioğlu et al. (P6) [28]	2022	High	High	Low	NA	High (94.4 mg/L)	High (31 mm/h)	Bilateral	NA
Bahçecioğlu et al. (P7) [28]	2022	High	High	Low	NA	High (19.4 mg/L)	High (39 mm/h)	Bilateral	NA
Bahçecioğlu et al. (P8) [28]	2022	High	High	Low	NA	High (48.7 mg/L)	High (35 mm/h)	Bilateral	NA
Bahçecioğlu et al. (P9) [28]	2022	High	High	Low	NA	High (22.5 mg/L)	High (71 mm/h)	Unilateral (the left lobe)	NA
Bahçecioğlu et al. (P10) [28]	2022	High	High	Low	NA	High (7.6 mg/L)	High (46 mm/h)	Bilateral	NA
Bahçecioğlu et al. (P11) [28]	2022	High	High	Low	NA	High (26 mg/L)	High (50 mm/h)	Bilateral	NA
Bahçecioğlu et al. (P12) [28]	2022	High	High	Low	NA	High (67 mg/L)	High (50 mm/h)	Bilateral	NA
Álvarez Martín et al. [29]	2020	NA	High (2.18 ng/dL)	Low (0.11 mIU/L)	Positive	NA	High (68 mm/hr)	Enlarged thyroid with heterogeneous echotexture	Reduced uptake
Abreu et al. (P1) [30]	2021	NA	NA	NA	NA	NA	NA	Hypoechogenic area in the right thyroid lobe	NA
Abreu et al. (P2) [30]	2021	NA	High (1.8 ng/dL)	NA	NA	NA	NA	Irregular and hypoechogenic area in the right thyroid lobe	NA
Abreu et al. (P3) [30]	2021	NA	NA	NA	NA	NA	NA	Solid hypoechogenic nodule in left thyroid lobe	NA
Asfuroglu et al. [19]	2020	High (7.7 pmol/L)	High (25.7 pmol/L)	Low (<0.008 mIU/L)	NA	High (101 mg/L)	High (134 mm/h)	Heterogenous thyroid with diffuse decrease of vascularity	NA
Ashraf et al. [31]	2021	Normal range	High	Low	NA	High	High	NA	NA
Asimi & Dzuvo [32]	2021	High	High	Low	Negative	NA	NA	Enlarged thyroid with diffuse and bilateral hypoechoic areas and absent vascularization	Reduced uptake
Barahona et al. [33]	2021	Normal (3.21 ng/mL)	High (1.83 pg/ml)	Low (0.2 IU/l)	Negative	High (10 mg/dl)	High (58 mm/h)	NA	Increased uptake
Brancatella et al. [34]	2021	High	High	Low	NA	High	High	Increased thyroid volume	NA
Brancatella et al. (P1) [17]	2020	High (8.0 pmol/L)	High (29.3 nmol/L)	Low (0.1 mU/L)	Negative	High (11.2 mg/L)	High (74 mm/h)	Enlarged thyroid gland with multiple hypoechoic areas	NA
Brancatella et al. (P2) [17]	2020	High (8.9 pmol/L)	High (31.8 nmol/L)	Low (<0.01 mU/L)	Negative	High (7.9 mg/L)	High (110 mm/h)	Increased thyroid gland with multiple hypoechoic areas and low vascularization	No uptake
Brancatella et al. (P3) [17]	2020	NA	NA	NA	NA	NA	NA	Increased thyroid gland with multiple hypoechoic areas and absent vascularization	NA
Brancatella et al. (P4) [17]	2020	High (6.9 pmol/L)	High (27.8 nmol/L)	Low (<0.01 mIU/L)	NA	High (8 mg/L)	NA	Increased thyroid gland with multiple hypoechoic areas	NA
Brancatella et al. [35]	2020	High (8.7 pmol/L)	High (27.2 nmol/mL)	Low (<0.04 mIU/L)	Negative	High (6.9 mg/L)	High (90 mm/h)	Multiple diffuse hypoechoic areas	NA
Campos-Barrera et al. [2]	2020	High (211 ng/dL)	High (1.6 ng/dL)	Low (undetectable)	Negative	High (66 mg/L)	High (72 mm/hr)	NA	No uptake
Chakraborty et al. [36]	2020	High (2.88 ng/mL)	High (20.1 μg/dL)	Low (<0.005 mIU/L)	Negative	High (16.6 mg/L)	High (110 mm/h)	Diffuse bilateral enlargement of the thyroid with hypoechogenicity and increased vascularity	Reduced uptake
Chong et al. [37]	2021	High (202 ng/dL)	High (2.3 ng/dL)	Low (0.01 mIU/L)	Negative	High (14 mg/L)	High (31 mm/h)	Diffusely heterogeneous echotexture	NA
Das & Fatima [38]	2021	NA	High (3.84 ng/dL)	Low (0.001 mIU/L)	NA	NA	High (80 mm/h)	NA	Reduced uptake
Davoodi et al. [39]	2021	High (236 ng/dL)	High (23.1 μg/dL)	Low (<0.001 mIU/L)	Negative	High (37.9 mg/L)	High (84 mm/h)	Heterogeneous thyroid gland with bilateral ill-defined hypoechoic areas	NA
de la Higuera López-Frías et al. [40]	2021	High (2.57 nmol/L)	High (27.93 pmo/L)	Low (0.008 mIU/L)	Negative	High (1.05 mg/dL)	High (31 mm/h)	Diffuse bilateral hypoechoic areas and low or absent vascularization	Reduced uptake
De San Juan et al. [41]	2020	Normal (1.4 ng/mL)	Normal (1.68 pg/mL)	Low (0.05 mIU/L)	Negative	High (5.09 mg/dL)	NA	Heterogenous thyroid tissues with normal vascularity	NA
Dworakowska et al. [42]	2021	High (8.5 pmol/L)	High (23.4 pmol/L)	Low (<0.01 mIU/L)	Positive	High (15.3 mg/L)	High (22 mm/h)	Patchy areas of variably reduced parenchymal echogenicity bilaterally	Reduced uptake
Feghali et al. [43]	2021	NA	High (1.9 ng/dL)	Low (0.01 mIU/L)	Positive	NA	NA	NA	Reduced uptake
Ippolito et al. [44]	2020	High (5.5 pg/mL)	High (24.6 pg/mL)	Low (0.08 mIU/L)	Negative	NA	NA	Enlarged hypoechoic thyroid, decreased vascularity	No uptake
Khatri et al. [45]	2021	High (3.39 nmol/L)	High (60.63 pmol/L)	Low (0.01 mU/L)	Positive	High (36.4 mg/L)	High (107 mm/h)	Heterogenous thyroid gland, with bilateral patchy ill-defined hypoechoic areas	NA
Mathews et al. [46]	2021	NA	High (2.1 ng/dL)	Low (0.029 mU/L)	Negative	High (5.3 mg/dL)	High (37 mm/h)	Mildly enlarged thyroid gland with no increased vascularity and 5-mm bilateral cysts	NA
Mattar et al. [47]	2020	High (13.4 pmol/L)	High (41.8 pmol/L)	Low (<0.01 mU/L)	Negative	High (122 mg/L)	NA	Enlarged thyroid gland with heterogenous echotexture, hypoechoic areas with ill-defined margins; reduced blood flow in both lobes	NA
Mehmood et al. [48]	2020	High (374 ng/L)	High (4.4 ng/L)	Low (0.01 mIU/L)	Negative	High (44 mg/L)	High (84 mm/h)	Heterogeneously enlarged thyroid gland	NA
Osorio Martínez et al. [49]	2021	High (643.4 ng/dl)	High (12.0 μg/dl)	Low (0.01 mIU/L)	Negative	NA	NA	Diffusely enlarged micronodular thyroid gland	No uptake
Ramsay et al. [50]	2021	NA	High (1.90 ng/dL)	Low (0.00004 mIU/L)	NA	NA	NA	NA	NA
Seyed Resuli & Bezgal (P1) [51]	2021	High (8.4 pmol/L)	High (26.8 pmol/L)	Low	Negative	High	High (65 mm/h)	Bilateral hypoechoic areas in the thyroid gland	Reduced uptake
Seyed Resuli & Bezgal (P2) [51]	2021	High (9.6 pmol/L)	High (28.1 pmol/L)	Low	Negative	High	High (58 mm/h)	Diffuse hypoechoic growth and reduced vascularization in the right thyroid lobe	Reduced uptake
Seyed Resuli & Bezgal (P3) [51]	2021	High (14.2 pmol/L)	High (43.1 pmol/L)	Low	Negative	High	High (70 mm/h)	Diffuse large and hypoechogenic thyroid	No uptake
Seyed Resuli & Bezgal (P4) [51]	2021	High (11.3 pmol/L)	High (38.1 pmol/L)	Low	Negative	High	High (65 mm/h)	Hypoechogenic thyroid	No uptake
Seyed Resuli & Bezgal (P5) [51]	2021	High (16.2 pmol/L)	High (43.5 pmol/L)	Low	Negative	High	High (80 mm/h)	Diffuse growth and hypoechoic appearance in the left thyroid lobe	Reduced uptake
Ruano et al. [52]	2020	NA	High (37.5 pmol/L)	Low (< 0.001 mU/L)	Negative	High (176 mg/L)	High (116 mm/h)	NA	No uptake
Ruggeri et al. [18]	2020	High (7.03 pg/mL)	High (2.69 ng/dL)	Low (0.006 mU/L)	Negative	High (8.8 mg/L)	High (60 mm/h)	Diffusely enlarged and hypoechogenic thyroid gland	Reduced uptake
Sohrabpour et al. (P1) [53]	2021	High (18.9 pmol/L)	Normal (19.5 pmol/L)	Low (0.07 mIU/L)	NA	High (28 mg/L)	High (70 mm/h)	Bilateral hypoechoic areas in the thyroid gland	NA
Sohrabpour et al. (P2) [53]	2021	High (25.4 pmol/L)	Low (2.3 pmol/L)	Low (<0.01 mIU/L)	NA	High (38 mg/L)	High (56 mm/h)	Bilateral hypoechoic areas in the thyroid gland	NA
Sohrabpour et al. (P3) [53]	2021	High (19.3 pmol/L)	High (24.7 pmol/L)	Low (0.12 mIU/L)	NA	High (18 mg/L)	High (45 mm/h)	Bilateral hypoechoic areas in the thyroid gland	NA
Sohrabpour et al. (P4) [53]	2021	High (23.7 pmol/L)	High (21.9 pmol/L)	Low (<0.01 mIU/L)	NA	High (43 mg/L)	High (83 mm/h)	Bilateral hypoechoic areas in the thyroid gland	NA
Sohrabpour et al. (P5) [53]	2021	High (21.6 pmol/L)	High (26.7 pmol/L)	Low (0.17 mIU/L)	NA	High (51 mg/L)	High (76 mm/h)	Bilateral hypoechoic areas in the thyroid gland	NA
Sohrabpour et al. (P6) [53]	2021	High (18.1 pmol/L)	Normal (18.4 pmol/L)	Low (0.23 mIU/L)	NA	High (23 mg/L)	High (39 mm/h)	Bilateral hypoechoic areas in the thyroid gland	NA
Sato et al. [54]	2021	High (7.3 pg/mL)	High(3.2 ng/dL)	Low (0.0 mIU/L)	Negative	High (3.6 mg/dL)	High (93 mm/h)	Diffuse hypoechoic areas	No uptake
Semikov et al. (P1) [55]	2021	NA	High (3.2 ng/dL)	Low (0.005 mIU/L)	Negative	High (3.32 mg/dL)	High (32 mm/h)	Diffuse enlargement of the thyroid gland	NA
Semikov et al. (P2)	2021	NA	High (3.4 ng/dL)	Low (0.0083 mIU/L)	Negative	High (0.61 mg/dL)	High (47 mm/h)	Diffuse inhomogeneous echostructure due to multiple hypoechoic areas	NA
Sherpa et al. [56]	2021	High (5.24 pg/mL)	High (3.0 ng/dL)	Low (0.182 mIU/L)	NA	NA	NA	Normal-sized gland, few sub-centimeter nodules bilaterally	Reduced uptake
Stasiak et al. (P1) [57]	2021	Normal (3.75 pg/mL)	Normal (0.96 ng/dL)	Normal (1.53 mIU/L)	Negative	High (9 mg/dL)	High (52 mm/h)	Several hypoechoic areas in the right thyroid lobe	NA
Stasiak et al. (P2) [57]	2021	High (21.6 pg/mL)	High (>7.7 ng/dL)	Low (<0.005 mIU/L)	Negative	High (60 mg/dL)	High (59 mm/h)	Several hypoechoic areas in the right thyroid lobe	NA
Stasiak et al. (P3) [57]	2021	High (5.13 pg/mL)	High (2.39 ng/dL)	Low (0.01 mIU/L)	Negative	High (4.98 mg/dL)	High (140 mm/h)	Several hypoechoic areas in both lobes	NA
Stasiak et al. (P4) [57]	2021	High (5.24 pg/mL)	High (2.34 ng/dL)	Low (0.07 mIU/L)	Negative	High (4.71 mg/dL)	High (117 mm/h)	Several hypoechoic areas in both lobes	NA
Thimmaiah et al. [58]	2021	High (4.7 pg/mL)	High (1.96 ng/dL)	Low (0.02 mIU/L)	NA	NA	High (55 mm/h)	NA	No uptake
Tjønnfjord et al. [59]	2021	High (7.5 pmol/L)	High (27.8 pmol/L)	Low (0.01 mIU/L)	Negative	High (86 mg/dL)	High (92 mm/h)	Slight diffuse enlargement of the thyroid gland	NA
Mondal et al. [60]	2022	High (82%)	High (82%)	Low	Negative (72.7%)	High	High	Diffusely enlarged thyroid gland, patchy hypoechoic areas	Reduced uptake (63.6%)

All patients were managed conservatively with non-steroidal anti-inflammatory drugs (NSAIDs) (aspirin, ibuprofen, and mefenamic acid) in 17 (16.5%) and steroids in 52 (50.5%). The steroids used included prednisone (15-60 mg), prednisolone (1-35 mg), methylprednisolone (16-40 mg), and dexamethasone (4 mg). Steroids and NSAIDs combination were given in nine patients, and in 12 (11.6%) patients, beta blockers were added in the form of 20-40 mg of propranolol. No treatment was administered in seven cases. Complete resolution of symptoms was achieved in 72% of cases. Subclinical hypothyroidism was observed in 25 patients and there was a recurrence of SAT symptoms in one case (Table 4).

**Table 4:** Clinical management and illness outcome of patients with COVID-19-associated SAT. SAT: subacute thyroiditis; NSAIDs: non-steroidal anti-inflammatory drugs; NA: not applicable.

Author	Year	Clinical management	Outcome
Bahçecioğlu et al. (P1) [28]	2022	Methylprednisolone (24 mg for 6 weeks, tapering regimen)	Complete resolution
Bahçecioğlu et al. (P2) [28]	2022	Methylprednisolone (24 mg for 6 weeks, tapering regimen)	Complete resolution
Bahçecioğlu et al. (P3) [28]	2022	Methylprednisolone (32 mg for 6 weeks, tapering regimen)	Complete resolution
Bahçecioğlu et al. (P4) [28]	2022	NSAID (3 weeks)	Complete resolution
Bahçecioğlu et al. (P5) [28]	2022	NSAID (2 weeks), methylprednisolone (24 mg for 5 weeks, tapering regimen)	Complete resolution
Bahçecioğlu et al. (P6) [28]	2022	NSAID (1 week), methylprednisolone (16 mg for 4 weeks, tapering regimen)	Complete resolution
Bahçecioğlu et al. (P7) [28]	2022	NSAID (4 weeks)	Complete resolution
Bahçecioğlu et al. (P8) [28]	2022	NSAID (1 week), methylprednisolone (32 mg for 7 weeks, tapering regimen)	Complete resolution
Bahçecioğlu et al. (P9) [28]	2022	No treatment	Complete resolution
Bahçecioğlu et al. (P10) [28]	2022	Methylprednisolone (32 mg for 6 weeks, tapering regimen)	Complete resolution
Bahçecioğlu et al. (P11) [28]	2022	Methylprednisolone (16 mg for 4 weeks, tapering regimen)	Complete resolution
Bahçecioğlu et al. (P12) [28]	2022	Methylprednisolone (32 mg for 36 weeks, tapering regimen), colchicine (1 mg for 9 weeks)	Complete resolution
Álvarez Martín et al. [29]	2020	Prednisone (40 mg/day as the starting dose, gradually tapered for 6 weeks)	Mild hypothyroidism
Abreu et al. (P1) [30]	2021	Prednisone at 15 mg/day	Complete resolution after 4 days
Abreu et al. (P2) [30]	2021	Prednisone at 15 mg/day	Complete resolution after 18 days
Abreu et al. (P3) [30]	2021	NA	Complete resolution after 2 weeks
Asfuroglu et al. [19]	2020	Hydroxychloroquine tablet 200 mg bid for 5 days, and prednisolone 16 mg daily tapering regimen	NA
Ashraf et al. [31]	2021	Dexamethasone (4 mg BD) and naproxen were initiated. After 3 days, oral prednisolone (15 mg BD) was given (tapering regime) for a week	Complete resolution after 2 weeks
Asimi & Dzuvo [32]	2021	Aspirin in 3 patients, prednisone in 2 patients, paracetamol in 3 patients, and ibuprofen in 3 patients (propranolol for tachycardia and tremors)	Complete resolution after 10 weeks (8 patients were euthyroid, whereas 3 were with subclinical hypothyroidism)
Barahona et al. [33]	2021	No specific antithyroid treatment (propranolol was started and later stopped in the absence of symptoms)	Complete resolution
Brancatella et al. [34]	2021	16 were on steroids, 1 on NSAIDs, and 1 with no treatment	13 had hypothyroidism at 3 months
Brancatella et al. (P1) [17]	2020	Prednisone therapy (25 mg/d) was started	Complete resolution
Brancatella et al. (P2) [17]	2020	Prednisone (25 mg/day), propranolol 40 mg/day	Subclinical hypothyroidism
Brancatella et al. (P3) [17]	2020	Ibuprofen 600 mg/day	Subclinical hypothyroidism
Brancatella et al. (P4) [17]	2020	Prednisone (25 mg/day)	Complete resolution
Brancatella et al. [35]	2020	Prednisone (25 mg/d) tapering regimen	Complete resolution
Campos-Barrera et al. [2]	2020	No treatment	Complete resolution
Chakraborty et al. [36]	2020	Prednisolone (30 mg/day as the starting dose, gradually tapered), propranolol 40 mg/day	Hypothyroid 1 month after follow-up
Chong et al. [37]	2021	Oral aspirin for his neck pain, and propranolol for tachycardia and tremors	Hypothyroidism after 3 weeks
Das & Fatima [38]	2021	Flurbiprofen and propranolol were initiated but symptoms did not improve. Tapering doses of prednisolone were started	After a few days of starting steroids, his symptoms resolved
Davoodi et al. [39]	2021	Dexamethasone 4 mg every 8 hours for 5 days, and then oral prednisone 25 mg daily	Complete resolution
de la Higuera López-Frías et al. [40]	2021	Ibuprofen 600 mg, propranolol 10 mg, and omeprazole 20 mg	Complete resolution after 15 days
De San Juan et al. [41]	2020	Mefenamic acid was started but was later shifted to celecoxib due to epigastric pains	Hypothyroidism after 8 weeks
Dworakowska et al. [42]	2021	Simple analgesics and propranolol	Sub-clinical hypothyroidism after 6 weeks
Feghali et al. [43]	2021	No treatment	Hypothyroidism after 3 weeks
Ippolito et al. [44]	2020	40 mg intravenous methylprednisolone for 3 days, then continuing with 25 mg oral prednisone	Complete resolution
Khatri et al. [45]	2021	Oral ibuprofen 600 mg every 6 hours and prednisone 40 mg daily	Complete resolution
Mathews et al. [46]	2021	Prednisone 20 mg daily, methimazole 10 mg daily (tapering regimen)	Complete resolution after 11 months
Mattar et al. [47]	2020	Prednisolone at a dose of 20 mg, and then gradually tapered	Complete resolution
Mehmood et al. [48]	2020	Prednisone (20 mg) and atenolol (25 mg) daily	Complete resolution
Osorio Martínez et al. [49]	2021	Atenolol 50 mg twice daily and prednisone 50 mg with gradual dosage tapering over 2 weeks	Complete resolution
Ramsay et al. [50]	2021	Ibuprofen initially. Propranolol 20 mg twice daily for 2 weeks	Complete resolution after 17 days
Seyed Resuli & Bezgal (P1) [51]	2021	Aspirin	Complete resolution after 15 days
Seyed Resuli & Bezgal (P2) [51]	2021	Aspirin	Complete resolution after 25 days
Seyed Resuli & Bezgal (P3) [51]	2021	Prednisolone (1 mg/kg)	Complete resolution after 29 days
Seyed Resuli & Bezgal (P4) [51]	2021	Aspirin	Complete resolution after 22 days
Seyed Resuli & Bezgal (P5) [51]	2021	Prednisolone (1 mg/kg)	Complete resolution after 34 days
Ruano et al. [52]	2020	500 mg of aspirin and 40 mg of propranolol every 6 hours	Complete resolution
Ruggeri et al. [18]	2020	Oral Prednisone (25 mg/day as the starting dose)	Complete resolution
Sohrabpour et al. (P1) [53]	2021	Prednisolone 25 mg/day	Complete resolution
Sohrabpour et al. (P2) [53]	2021	Prednisolone 25 mg/day	Complete resolution
Sohrabpour et al. (P3) [53]	2021	Prednisolone 25 mg/day	Complete resolution
Sohrabpour et al. (P4) [53]	2021	Prednisolone 25 mg/day	Complete resolution
Sohrabpour et al. (P5) [53]	2021	Prednisolone 25 mg/day	Complete resolution
Sohrabpour et al. (P6) [53]	2021	Prednisolone 25 mg/day	Complete resolution
Sato et al. [54]	2021	NSAIDs for three days, then oral prednisolone (15 mg/day as the initial dose, tapering regimen)	Complete resolution after 7 weeks
Semikov et al. (P1) [55]	2021	Prednisone 30 mg per day for 4 weeks, tapering regimen	Complete resolution after 30 days
Semikov et al. (P2) [55]	2021	Prednisone 30 mg per day for 4 weeks, tapering regimen	Complete resolution after 30 days
Sherpa et al. [56]	2021	No treatment	Complete resolution after a week
Stasiak et al. (P1) [57]	2021	Prednisone started, tapering regimen	Complete resolution after 10 months of follow-up
Stasiak et al. (P2) [57]	2021	Prednisone 60 mg daily	Complete resolution after 4 months
Stasiak et al. (P3) [57]	2021	Prednisone started	SAT recurrence
Stasiak et al. (P4) [57]	2021	Prednisone 40 mg daily, tapering regimen	Complete resolution
Thimmaiah et al. [58]	2021	No treatment for SAT	NA
Tjønnfjord et al. [59]	2021	Prednisolone 20 mg daily for 1 month	Complete resolution after 2 months
Mondal et al. [60]	2022	Beta-blockers = 11, NSAIDs = 2, glucocorticoid = 4	Complete resolution (n = 9) and hypothyroidism (n = 2)

The true prevalence of thyroid disease and thyroid function abnormalities in COVID-19 is largely unknown. Given the fact that only a few reports of SAT have appeared against the backdrop of more than 500 million reported cases of COVID-19 worldwide, SAT seems to be a rather rare manifestation of COVID-19 infection. The probable reason may be either SAT is being misdiagnosed with COVID-19 symptoms, or there is masking of SAT symptoms due to routine use of corticosteroids and NSAIDs during COVID-19 management. According to Trimboli et al. [25], SAT cannot be considered a direct or frequent complication of SARS-CoV-2. A study comparing SAT cases during the COVID-19 pandemic to those observed in the previous years reported that the COVID-19 pandemic influenced the severity of the disease, leading to more severe forms of the disease, although COVID-19 was not associated with a rise in the overall number of SAT cases in 2020 compared to the previous years [34]. The thyroid gland may be vulnerable to COVID-19 infection due to the abundance of angiotensin-converting enzyme 2 (ACE2) receptors in the thyroid parenchyma, which might affect thyroid function. Serum levels of TSH, T3, and T4 in COVID-19 patients were found to be significantly lower than those in the control group, and a positive correlation between the severity of SARS and T3 levels was reported [61-63].

After the first report of SAT with COVID-19 infection in an 18-year-old female in Italy by Brancatella et al. [35], several such cases have been reported worldwide. These reports suggest that SARS-CoV-2 infection may serve as a probable trigger for the development of SAT, either during the active infection or even after the acute illness has resolved. To date, only three systematic studies on a similar topic have been conducted, with 27 (17 case reports and two case series) [25], 21 (11 case reports and two case series) [64], and 17 cases [65] with SAT. Among these, the latest systematic review retrieved studies until 20th April 2021. In these reviews, only case reports and case series were available, and the number and quality of published data on SAT in COVID-19 patients were rather inadequate. We have conducted the most comprehensive and up-to-date systematic review of the published literature and have included all reported cases till the date of review, identifying 103 SAT cases in 37 included articles with a more detailed presentation of data. Our review demonstrates that SAT is an uncommon association of SARS-CoV-2 viral illness, which may occur either concomitant with the illness or even after recovery from the primary viral illness, presents with mild to moderate symptoms, and generally responds to conservative medical therapy. Table 5 gives the overall summary of the findings of this systematic review.

**Table 5:** Clinico-sociodemographic profile of included cases with COVID-19-associated SAT. SAT: subacute thyroiditis; CRP: C-reactive protein; ESR: erythrocyte sedimentation rate; TSH: thyroid-stimulating hormone; T4: tetraiodothyronine; T3: triiodothyronine; TPO-Ab: thyroid peroxidase antibody; COVID-19: coronavirus disease 2019; RT-PCR: reverse transcription polymerase chain reaction; NSAIDs: non-steroidal anti-inflammatory drugs; NA: not applicable.

Variable		N	%
Total		103	100.0
Age range	18-73 years		
Gender	Male	24	23.3
	Female	79	76.7
Continent	Asia	43	41.7
	Europe	47	45.6
	North America	10	9.7
	South America	3	2.9
COVID-19 diagnosis	Positive IgG	9	8.7
	RT-PCR	74	71.8
	Not mentioned	22	21.4
Onset of SAT symptoms	Concurrent	8	7.8
	1-30 days	54	52.4
	>30	19	18.4
	No information	20	19.4
Presence of goiter	YES	20	19.4
	NO	54	52.4
	No information	29	28.2
Presence of general symptoms of SAT	YES	93	90.3
	NO	10	9.7
Neck pain	YES	86	83.5
	NO	17	16.5
Fever	YES	71	68.9
	NO	32	31.1
Palpitations	YES	50	48.5
	NO	53	51.5
Tremors	YES	25	24.3
	NO	78	75.7
T3	High	85	82.5
	Low	1	1.0
	Normal	5	4.9
	No information	12	11.7
T4	High	93	90.3
	Low	1	1.0
	Normal	6	5.8
	No information	3	2.9
TSH	Low	98	95.1
	Normal	1	1.0
	No information	4	3.9
TPO-Ab	Positive	7	6.8
	Negative	49	47.6
	No information	47	45.6
CRP (mg/L)	High	80	77.7
	No information	23	22.3
ESR (mm/h)	High	78	75.7
	Normal	2	1.9
	No information	23	22.3
Ultrasonography	Confirming SAT	95	92.2
	No information	8	7.8
Thyroid scan	Increased uptake	1	1.0
	No/reduced uptake	38	36.9
	No information	64	62.1
Treatment	NSAID	17	16.5
	Steroid	52	50.5
	Beta-blocker	5	4.9
	NSAID + steroid	9	8.7
	Steroid + beta-blocker	8	7.8
	NSAID + beta blocker	4	3.9
	No treatment	7	6.8
	No information	1	1.0
Outcome of illness	Complete resolution	74	71.8
	Recurrence of SAT	1	1.0
	Hypothyroidism	25	24.3
	No information	3	2.9

Studies have reported that SAT is more prevalent in females than males (4:1 ratio) and most often occurs at 40 to 50 years of age [37]. We observed that reported cases were more common in females (female-to-male ratio of 3:1) with a median age of 41 years at presentation. The incidence of SAT is higher in females compared to males (19.1 vs. 4.1 per 100,000/year, respectively) [14]. This can partly be explained by greater levels of ACE-2 and TMPRSS2 expression in women [66-68]. These circumstances may explain why SARS-CoV-2 causes SAT more commonly in women [66]. Men, on the other hand, are more vulnerable to COVID-19 than women and have a poorer prognosis [69].

In a prospective study, the clinical features of COVID-19-associated SAT patients were similar to those of classical SAT [28]. Our systematic review demonstrates that clinical characteristics, laboratory results, and therapy characteristics of SAT with COVID-19 are similar to those described in classical SAT. Temporally, SAT developed following the COVID-19 infection in most cases, and concurrently only in a few, with clinical symptoms such as neck pain, fever, palpitations, and tremors. According to the most recent systematic review [25], the time between COVID-19 and the onset of SAT symptoms ranged from three to 60 days, and patients included in our review had a median onset time of 28 (IQR: 12-36) days. Wang et al. [70] reported that T3, T4, and TSH levels among patients with SARS-CoV-2 were considerably lower than those in the control group but here low or undetectable TSH and high T3 and T4 that may differentiate it from the sick euthyroid syndrome or non-thyroidal illness (NTI). Anti-TPO-antibody (reported negative in 48% of the patients), thyroid USG (revealing classic findings of hypoechoic glands), and thyroid scintigraphy (showing absent or reduced uptake) may serve to differentiate it from NTI of COVID-19 in some cases.

The course of SAT is usually self-limiting. The American Thyroid Association's (ATA) recommendations propose that therapy of SAT be guided by the severity of the condition, with steroids indicated for moderate to severe cases and NSAIDs for mild ones [71]. Accordingly, the cases have been managed variously with steroids and NSAIDS as the mainstay of treatment. Among the described cases, the majority achieved resolution of symptoms, i.e., 72% (95% CI: 62% to 80%); nevertheless, 25 patients (24%) had subclinical hypothyroidism at follow-up. Although the majority of cases return to euthyroidism, thyroid function needs to be closely monitored for at least six months after the resolution of symptoms.

Since the incidence and diversity of SAT in COVID-19 patients are undetermined, increased numbers of cases would be required to show its causation and relationship to the virus. Due to the rarity of cases, the causal relationship between SAT and COVID-19 cannot be established. However, recurrence of SAT may need longer follow-up since the rate of SAT recurrence has been found to be significantly higher in genetically susceptible people (especially with HLA-B18 and HLA-B35 haplotypes) [57]. Therefore, it may be worthwhile to observe if COVID-19 patients who develop SAT also have these HLA-B35 and HLA-B18 antigens.

With the drop in global occurrence of COVID-19 infection cases, we attempted to collate all the reported SAT and COVID-19 cases in addition to two of our cases till May 5, 2022. The aim is to have an updated description of all the data for understanding various aspects of the association. There are certain limitations in this study. First, this study largely included case reports and was thus vulnerable to selection bias. Second, it is likely that only clinically challenging cases were reported and published, therefore the propensity for publication bias. Third, it is quite possible that symptoms of SAT were misdiagnosed in severe COVID-19 disease or concealed, modulated/ameliorated by the routine administration of high-dose corticosteroids. Fourth, many cases have not given details on whether steroids were used as the first line of treatment, and some of the cases lacked details on clinical characteristics, laboratory results, and diagnostic parameters.

## Conclusions

SAT is a self-limited thyroid disease caused by a viral or post-viral SARS-CoV-2 infection. SAT can be considered another, although rare, extra-pulmonary clinical manifestation of COVID-19 infection, and its timely early detection and anti-inflammatory therapy can help the successful management of the disease. The index of suspicion of COVID-19-induced SAT should be elevated amongst clinicians, and treatment must be done on a case-by-case basis; however, corticosteroid therapy should be included in the treatment plan.
